# Differences in Online Consumer Behavior: A Multi-Dimensional Comparative Study in the Context of European Digital Commerce

**DOI:** 10.3390/bs15101384

**Published:** 2025-10-12

**Authors:** Radovan Madlenak, Roman Chinoracky, Natalia Stalmasekova, Lucia Madlenakova

**Affiliations:** Faculty of Operation and Economics of Transport and Communications, University of Zilina, Univerzitná 8215/1, 010 26 Žilina, Slovakia; roman.chinoracky@uniza.sk (R.C.); natalia.stalmasekova@uniza.sk (N.S.); lucia.madlenakova@uniza.sk (L.M.)

**Keywords:** cross-cultural differences, consumer perceptions, determinants of online shopping, e-commerce, online shopping behavior

## Abstract

The aim of this study was to analyze international differences in online consumer behavior. The analysis was carried out on a sample of 763 participants from the countries of Spain, France, Poland and Russia. Online consumer behavior was examined from the perspective of seven dimensions: shipping-related concerns and preferences, price sensitivity and perceived cost advantage, quality perception, security concerns, time-related benefits, availability and quality of information, and shopping service satisfaction. Data were verified using Average inter-item correlation, the Shapiro–Wilk test and Levene Statistic. Subsequently, Welch’s ANOVA and one-way ANOVA and the Games–Howell and Tukey HSD post hoc tests were applied. Statistically significant differences were fully identified in all examined dimensions. The largest differences were recorded in price sensitivity, shipping-related concerns and security concerns. The effect measurements, in addition to ANOVA and post hoc tests, confirm the significance of these differences. National context, shaped by culture, institutional trust and digital infrastructure, continues to influence online consumer behavior. The strategies that the businesses should adopt should focus on approaches that are tailor-made for a specific market. This means that adapting pricing models, strengthening trust (e.g., through secure payments and strengthening safe return policies), and adapting delivery options to local preferences can lead to improved customer satisfaction in cross-border e-commerce.

## 1. Introduction

Empirical research has examined various factors affecting online shopping behavior, including perceived product quality, pricing, security, convenience, access to product information, and the quality of delivery and after-sales services (Vasić et al., 2019; Kaurin & Bošković, 2020; Huterska & Huterski, 2022; Radionova-Girsa et al., 2019; Svatošová & Dvorský, 2025; Pham & Ahammad, 2017). Results of studies conclude that online consumer behavior reflects an interplay of economic factors, institutional trust, cultural expectations, and prior experiences with digital technologies.

Although European regulations, harmonized market standards, and shared technological infrastructures exist across parts of the continent, previous research indicates persistent variation in consumer trust, perceived risk, service expectations, and price sensitivity across European markets. Many studies have identified differences in delivery-related concerns (Vasić et al., 2021; Majchrzak-Lepczyk, 2019; WIK-Consult, 2021; Kim et al., 2017), price sensitivity and fairness perceptions (Baye et al., 2002; Cardona et al., 2015; Hinz et al., 2011; Kemper, 2018; D’Adamo et al., 2021), perceptions of product quality (Știr, 2018; Pawełoszek & Bajdor, 2020; Gracz, 2024; Stoklasa & Halaška, 2025; Hunter, 2024), security concerns (Pavlou & Gefen, 2004; D’Adamo et al., 2021; Nyshadham & Ugbaja, 2006; Jiang, 2025), time-related benefits (Q. Zhu & Semeijn, 2015; An et al., 2016; Devi & Kumari, 2015; Kyureghian & Caillavet, 2025), information reliability and digital literacy (W. Zhu et al., 2021; Macready et al., 2025; Daiya, 2023; Grant & Philipp, 2014; Huterska & Huterski, 2022), and customer satisfaction (Kemper, 2018; Kristensen & Johansson, 2008; Pedersen & Schmidt, 2011; Hunter, 2024; Sendcloud, 2022; Vasić et al., 2019). Findings suggest that even under harmonized regulatory frameworks, consumer perceptions and behaviors remain influenced by a combination of cultural, institutional, psychological, and infrastructural factors, which vary across countries and market contexts. Focus often targets a limited range of behavioral dimensions or a specific national market. Consumer behavior on a multinational scale, influenced by economic, institutional and cultural factors, remains under-researched. This study addresses this research gap by focusing on a multidimensional and multinational perspective combining both perspectives into broader context. Such context provides a basis for a better understanding of consumer behavior across Europe.

From a multidimensional perspective, the analysis focuses on seven dimensions: shipping-related concerns and preferences, price sensitivity and perceived cost advantages, product quality perception, security concerns, time-related benefits, information availability and quality, and shopping service satisfaction. Consumer attitudes regarding these dimensions were collected through questionnaire surveys. Data collection was conducted in a multinational perspective of Spain, France, Poland, and Russia. The selected samples of countries capture geographical, cultural, and economic diversity within Europe. For each dimension, seven corresponding hypotheses were formulated to examine cross-country differences in online shopping behavior.

A three-step methodological approach was applied to test hypotheses. In the first step, average inter-item correlation was used to assess internal reliability. In the second step, Levene’s test was used to evaluate the homogeneity of variances. In the third step, depending on the results of the second step, either one-way ANOVA or Welch’s ANOVA was applied and for pairwise comparisons Tukey’s HSD or Games–Howell tests were used.

The article is organized as follows: the literature review summarizes existing empirical studies and conceptual models related to online consumer behavior; the Section 3 describes the data collection process, sample characteristics, measurement instruments, and statistical procedures; the Section 4 presents the empirical findings; the Section 5 interprets these findings in the context of existing literature and outlines implications for practitioners and policymakers; and the Section 6 summarizes the entire study, states limitations and suggests directions for future research.

## 2. Literature Review

Various authors have researched determinants of consumer choice in the case of online shopping in European markets. Vasić et al. (2019), Kaurin and Bošković (2020), Huterska and Huterski (2022), Radionova-Girsa et al. (2019), Svatošová and Dvorský (2025), and Pham and Ahammad (2017) have all examined various determinants that condition online consumer choice. The following summary discusses each of these studies individually to reflect the diversity of factors identified.

Vasić et al. (2019), in the case of Serbia, recognized some determinants of consumer satisfaction as security, information availability, shipping conditions, product quality, prices, and time factors. Kaurin and Bošković (2020), in their research on the Serbian market as well, discovered five dimensions: website quality, availability of information, security, privacy, and reliability.

Huterska and Huterski (2022) used Eurostat data to investigate broader determinants, setting technological determinants (e.g., access to the internet), demographic factors (e.g., age, educational level, working in science and research), and economic factors (e.g., GDP per capita) as relevant for the behavior of online shopping.

Radionova-Girsa et al. (2019) proposed a broader set of determinants, including past positive experience, communication with sellers, buyers’ opinion, social media and influencers, word of friends, loyalty programs, product quality and looks, site usability, safety, delivery speed, price advantages, and consumer motivation (hedonic versus utilitarian). They relied on Eurostat data.

Based on Czech respondents, Svatošová and Dvorský (2025) examined perceived usefulness, ease of use, trust, perceived risk, social influence, attitude towards online shopping, purchase intention, customer satisfaction, and e-loyalty.

Pham and Ahammad (2017), in studying the UK market, identified product information, customization facilities, fulfillment processes, and responsiveness of customer service as drivers of online shopping satisfaction.

Studies presented in this part reveal that consumer behavior on the Internet is influenced by a number of factors that can be divided into the following categories: (1) offer- and product-related factors, (2) environment- and platform-related factors, (3) logistics- and customer service-related factors, (4) social- and personal-related factors, and (5) macro-level context-related factors.

This study discusses selected categories, i.e., (1), (2), and (3). In its scope, analysis examines seven dimensions: (A) attitudes and shipping-related matters, (B) price sensitivity and perceived cost advantage, (C) perception of product quality, (D) security concerns, (E) time advantages, (F) availability and quality of information, and (G) shopping service satisfaction. Research in this study does not examine a formal theoretical model. The dimensions of consumer behavior are theoretically derived based on the overall construct of online satisfaction as defined in Vasić et al. (2019), with dimensions A to G being identified as contributors to ultimate satisfaction in the online shopping platform. Some dimensions concentrate on critical dimensions regarding transactional experience (shipping, price, information credibility, security, and satisfaction with services) and on pragmatic concerns, most of which would be most important to consumer choice and cross-border e-commerce. This set allows systematic comparative study by nations. Social influence, psychological characteristics, or macroeconomic conditions (4) and (5) are acknowledged but not included in this analysis.

There are also other valuable theoretical frameworks on consumer behavior beyond the scope of this study: such as Hofstede’s cultural dimensions (Taras et al., 2010), the technology acceptance model (King & He, 2006), or the unification theory of technology acceptance and use (Venkatesh et al., 2003).

### 2.1. Shipping-Related Concerns and Preferences

Majchrzak-Lepczyk (2019) separated factors of logistics service quality, such as delivery timeliness, reliability, and flexibility, in 12 European markets. Using consumer survey data, the study concluded that while these factors are well documented, their relative importance differs across markets. Furthermore, Grant and Philipp (2014) observed that business-to-consumer logistics service quality has varying effects on customer satisfaction and customer loyalty across France, Germany, and the United Kingdom and is linked to delivery feature-based expectations including exact time slots, traceability, and returns.

Vasić et al. (2021) emphasized perceived control over parameters of delivery, for example, deciding time and place of delivery, as a driver of satisfaction with e-commerce logistics among customers. Uvet et al. (2024) discussed a hybrid model of the quality of e-logistics indicating that Northern Europeans are more interested in return procedures and real-time tracking compared to Southern Europeans. Kim et al. (2017) argued that geographic and distance-related logistics problems are causes of dissatisfaction in EU cross-border trade.

Industry analyses provided further insights. The Sendcloud (2022) European e-commerce survey, which was carried out among more than 7000 individuals, indicated that the Swiss customers were most sensitive to shipping costs (38%), while those of the Netherlands (22%) demonstrated relatively lower sensitivity. Italian respondents were more inclined to pay additional fees for swift delivery. Preferability of delivery formats varied too: 58% of French, 50% of Spanish, and 46% of Dutch consumers preferred pick-up/drop-off (PUDO) services. The WIK-Consult (2021) survey of over 17,000 respondents from 30 EU and EEA member states reported less satisfaction with cross-border delivery in Eastern European compared to Western European regions, especially for deliveries from outside the EU.

Ngo et al. (2025) reported that younger consumers, in this case Generation Z, value social responsibility and transparency in the logistics operations, including green packaging and carbon-free delivery. Gajewska et al. (2020) discovered that delivery satisfaction is associated with the logistics infrastructure maturity and countries such as the Netherlands and Germany score higher in re-liability measures.

Research indicates that shipping issues (i.e., delivery cost, service reliability, speed, return process, and delivery methods) are not equally experienced by consumers in Europe. It varies in terms of trust, country infrastructure, and cultural similarity. Most of the earlier research was conducted on shipping factors within each nation-state. Comparative studies directly measuring cross-country variations in how consumers perceive shipping issues are relatively less. Hence, a hypothesis is framed:

**H(A):**  *There are statistically significant differences between European countries in consumer concerns related to delivery in online shopping.*

### 2.2. Price Sensitivity and Perceived Cost Advantage

Baye et al. (2002) observed that despite the introduction of the euro, price disparities persisted between countries in the Eurozone and those outside it. The study reported that both the best available prices and average prices for identical products varied by country.

Consumer-focused research provides further insights. Hinz et al. (2011), in a study conducted in Germany, found that online demand declined sharply when a retailer’s listing appeared lower on price comparison platforms, particularly among first-time buyers, indicating sensitivity to price positioning. Kemper (2018), analyzing e-commerce transactions in the fashion sector across six European countries, reported variation in price elasticity across borders, moderated by cultural dimensions such as individualism and long-term orientation.

Cross-national studies suggest that perceptions of pricing fairness and purchasing power are not uniform. While the euro currency enhanced price transparency Baye et al. (2007) found out that online price competition remained uneven across countries while price competition was influenced by differences in consumer search behavior and digital literacy. Cardona et al. (2015) found that perceptions of cost advantages are shaped by transaction-related factors such as perceived risk, product variety, and the complexity of cross-border transactions, which differ across EU Member States.

Data from the European Commission’s Digital Economy and Society Index (European Commission, 2022) indicate that countries with more developed digital infrastructures and higher levels of digital literacy tend to exhibit narrower cross-border e-commerce price differentials and greater use of price comparison tools.

The conclusion of these studies is that price sensitivity and perceived pricing advantages vary across European countries. Consumers are influenced by technological readiness, cultural attitudes toward value and fairness, and engagement with mechanisms that increase price transparency. Most existing studies examine pricing factors within individual national contexts and comparative analyses directly assessing cross-country differences in price sensitivity and perceived pricing advantages remain relatively limited. Based on this rationale, the following hypothesis is formulated:

**H(B):**  *There are statistically significant differences in perceived pricing advantages of online shopping between European countries.*

### 2.3. Quality Perception

Research in Romania by Știr (2018) identified mistrust in product quality as one of the barriers to online shopping. Romanian consumers frequently relied on customer reviews as the primary source of product quality assessment, whereas in traditional in-store settings, price remained the dominant criterion. This illustrates context-dependent differences in how consumers evaluate product quality.

Pawełoszek and Bajdor (2020) found that in Poland, online shopping frequency and consumer profiles predicted perceived product quality. More frequent online shoppers tended to report higher perceived quality, though initial perceptions varied across socio-demographic groups.

Research by Teleaba and Popescu (2020), using data from Eastern Europe, reported that while product quality remains important in offline retail, its role in fostering customer loyalty in online environments is reduced. Product variety and website usability appeared to have greater influence. This indicates that perceptions of product quality in online retail may be more dependent on contextual factors, particularly in less developed e-commerce markets.

Gracz (2024), analyzing Polish consumers through a Technology Acceptance Model (TAM) framework, emphasized that trust in the platform and its functionality was important for perceived quality, especially among first-time online shoppers. Similarly, Lunardo et al. (2025) found that in France and Italy, perceptions of product quality improved when products were marketed as organic and aligned with expectations regarding eco-labeling and brand authenticity.

Stoklasa and Halaška (2025) studied Czech consumers and found that quality certifications played an important role in shaping trust and perceived product quality, particularly in the online food sector. Hunter (2024) reported that in five European countries, perceived quality—especially for food and cosmetic products—was influenced more by ethical labels (e.g., animal welfare) than by pricing cues.

Research suggests that perceptions of product quality in online shopping are not uniform across European countries. Differences appear to reflect variations in cultural norms, e-commerce development, quality labeling practices, and prior experience with digital retail. Patterns, of studies dedicated to national markets and limited comparative analyses directly assessing cross-country differences in perceptions of online product quality, are again present. Based on this fact, the following hypothesis is formulated:

**H(C):**  *There are statistically significant differences in perceived product quality in online shopping between European countries.*

### 2.4. Security Concerns

By multi-criteria decision analysis D’Adamo et al. (2021) classified European countries into three groups in terms of sensitivity toward online security issues. Danish, Dutch, and Swedish consumers were more concerned about cybersecurity and put the highest priority on safe digital environments while making online purchases.

Cardona et al. (2015) put forward that perceived risk continues to drive cross-border e-commerce in the European Union even when product price and availability are favorable. Customers in countries with weaker regulatory mechanisms or lower institutional trust revealed greater reluctance to share sensitive information, such as credit card details, due to identity theft and misuse issues.

Nyshadham and Ugbaja (2006) found, through psychometric modeling, that by country, perceptions of risk related to online shopping, e.g., privacy invasion and identity theft, differ. One country’s risky behavior, e.g., buying online using a debit card, is habituated in another depending on familiarity with electronic financial systems and cultural suitability.

Pavlou and Gefen (2004) determined that institutional trust and perceived system security were more accurate predictors of willingness to engage in online transactions, particularly in less inter-personal trusting nations. Institutional mechanisms can be substitutes for inter-personal trust when it comes to influencing consumer action online.

Jiang (2025) explained that trust in computerized digital systems, such as AI-powered customer service and self-driving delivery, was affected by previous attitudes toward digital security. Consumers from Scandinavian countries demonstrated greater acceptance of these technologies, which could be due to more trust in digital infrastructures and regulatory institutions.

Literature shows that European consumers’ attitude towards online security risks differs across nations, subject to technological, institutional, and cultural forces. Trends of cross-national context analyses exist and henceforth comparative studies explicitly testing cross-country variations in cross-country perceptions of online security risks are limited. Based on this fact, the following hypothesis is formulated:

**H(D):**  *There are statistically significant differences in perceived online security risks between European countries.*

### 2.5. Time-Related Benefits

Q. Zhu and Semeijn (2015), in a study of Western European consumers from the United Kingdom and the Netherlands, found that time savings predicted behavioral intention in online grocery shopping. This contrasts with research from the United States, where consumers prioritize product quality and service-related attributes.

Cross-national research highlights further differences shaped by cultural norms and market structures. An et al. (2016) reported that convenience and economic efficiency motivated cross-border online shopping, though their relative importance varied across consumer segments and countries. Some European consumers placed greater emphasis on continuous access to online shops (“24/7 availability”), indicating that preferences for temporal flexibility differ between markets.

Devi and Kumari (2015) identified timesaving and convenience as recognized benefits of online shopping but emphasized that such perceptions are mediated by digital infrastructure quality and users’ familiarity with e-commerce platforms. These mediating factors vary across European countries.

Giménez-Nadal and Molina (2015), analyzing time-use patterns across European countries, provided contextual insights into how time is allocated between work and non-work activities. Their findings help explain why some populations may attribute greater value to time-efficiency in online shopping, particularly in cases involving commuting, caregiving responsibilities, or limited physical retail access in rural areas.

Kyureghian and Caillavet (2025), investigating the online grocery sector in France, found that time-saving functionalities such as one-click reordering and same-day delivery were valued by urban professionals and working parents.

From research findings it can be stated that timesaving is often identified as an advantage of online shopping, but its perceived value and influence on consumer behavior differ across European countries. Differences are shaped by differences in digital readiness, infrastructure accessibility, and socio-cultural expectations related to convenience.

Patterns of analyses dedicated to national context are present. This means that comparative analyses directly assessing cross-country differences in perceptions of time-saving advantages again remain limited. On basis of these findings following hypothesis is formulated:

**H(E):**  *There are statistically significant differences in perceived time-saving benefits of online shopping between European countries.*

### 2.6. Availability and Quality of Information

W. Zhu et al. (2021), in a cross-continental study including European respondents, identified variation in how consumers understood and valued product descriptions. In some markets, consumers regarded product information as reliable and current, while in others, particularly in emerging digital markets, concerns were raised about ambiguity and limited product awareness. Disparities in information clarity may persist even within identical digital storefronts.

Pawełoszek and Bajdor (2020), using data from Poland, found that more frequent online shoppers expressed greater satisfaction with the completeness and accuracy of product information. This perception developed over time with experience, indicating that in less mature e-commerce environments, trust in information may remain limited.

Cardona et al. (2015), in an EU-level study, identified inconsistent and unclear product information as an obstacle to the growth of cross-border e-commerce. The problem was especially relevant in markets with fewer localized language interfaces or less transparent return policies, both of which affected consumer trust in product representations.

Mahmood et al. (2014) argued that national levels of trust and economic stability, rather than technological capacity alone, influence perceptions of online product information. Consumers in high-trust societies were more likely to consider product descriptions timely and accurate.

Macready et al. (2025), in a cross-national survey involving 13 European countries, found that trust in product and food information was strongly influenced by social trust and institutional confidence. Scandinavian respondents expressed higher trust in information quality than respondents from Southern and Eastern Europe.

In the Polish market, Gracz (2024) emphasized that perceived usefulness and credibility of product information were important in the selection of online sellers. Return policy transparency, review mechanisms, and visual presentation were especially influential in cultures characterized by high uncertainty avoidance.

Stoklasa and Halaška (2025) examined Czech consumers and found that third-party verified quality labels had a positive effect on trust in product information. Institutional signaling may help reduce cross-country disparities in perceived information reliability.

Daiya (2023) reported that consumers in technologically mature Western European markets responded more positively to enriched product formats such as videos and augmented views, compared to consumers in emerging markets, where reliance on static text descriptions was more common.

Results show that perceptions of product information availability and quality differ among European nations. These variations are found to be influenced by cultural orientation, institutional confidence, and consumers’ prior experience with digital retailing settings. Considering the identified disparities in how consumers across Europe assess and engage with online product information, the following hypothesis is proposed:

**H(F):**  *There are statistically significant differences in perceived information availability and quality between European countries*

### 2.7. Shopping Service Satisfaction

Kemper (2018), analyzing fashion e-commerce transactions across six European countries, found that cultural dimensions such as power distance and individualism influenced how consumers perceived service quality and expressed satisfaction. These cultural factors were associated with differences in recommendation likelihood and enjoyment, even when services were identical.

Kristensen and Johansson (2008) highlighted cultural adaptation as a necessary factor to apply when comparing satisfaction between nations. Employing anchoring vignettes to correct for response style bias, they established that Nordic consumers were more satisfied than Southern European consumers under similar circumstances. This suggests that national and psychological factors influence how consumers interpret and express satisfaction.

Pedersen and Schmidt (2011), in research on digital wellbeing, observed that satisfaction with digital services, including e-commerce, relates to individual digital engagement and the strength of the digital ecosystem in each region. Their results indicate that satisfaction with online shopping depends on broader digital access and competence.

Findings indicate that satisfaction with online retailing services is not homogeneous across Europe. Instead, they indicate a combination of culture-based expectations, trust frameworks, and relative national digital infrastructure building. On the basis of these findings, which consistently point to national variation in how consumers experience and report satisfaction, we propose the following hypothesis:

**H(G):**  *There are statistically significant differences in customer satisfaction with online shopping between European countries.*

### 2.8. Summary of Literature Review

Scientific evidence on consumer behavior in the online setting demonstrates that all of the seven dimensions covered in this review have been researched to varying degrees and levels. Most of the reviewed research only focuses on specific national settings or discusses one area of behavior, which limits their comparative applicability.

Shipping matters research shows that customers differentiate the way in which they evaluate convenience, quality and reliability of delivery: Majchrzak-Lepczyk (2019) studied the quality of logistics services in 12 EU countries and found variation in perceived importance of punctuality, reliability and flexibility; Grant and Philipp (2014) illustrated how the features of delivery affect satisfaction differently for France, Germany and the United Kingdom; Vasić et al. (2021) and Uvet et al. (2024) showed that control of delivery details and returns procedures are valued differently by regions; Kim et al. (2017) highlighted geographical constraints in cross-border logistics; Sendcloud (2022) and WIK-Consult (2021) provided extensive evidence of national preference of transport differences; and Ngo et al. (2025) and Gajewska et al. (2020) have underlined generational and infrastructure satisfaction drivers.

Price sensitivity tests provide evidence of global and domestic differences in consumer response to the price of goods in online trade: Baye et al. (2002) found long-lasting country-specific price differentials even after the introduction of the euro; Baye et al. (2007) connected transparency in prices to asymmetrical competition in markets; Hinz et al. (2011) found high price sensitivity among German consumers; Kemper (2018) observed variation in price elasticity across six countries that were mediated by cultural factors; Cardona et al. (2015) reported contextual determinants such as transaction complexity and perceived risk; European Commission (2022) evidence shows that advanced digital infrastructure weakens price variations.

Product quality perception studies confirm views that consumer quality perception is based on contextual and national factors: Știr (2018) established Romanian consumers’ reliance on reviews; Pawełoszek and Bajdor (2020) found that purchase frequency and demographic shopper characteristics affect perceptions of quality in Poland; Teleaba and Popescu (2020) found that usability rather than quality affects loyalty in Eastern Europe; Gracz (2024) underlined Polish consumers’ dependence on platforms; Lunardo et al. (2025) found that the “organic” label increased perceived quality in France and Italy; Stoklasa and Halaška (2025) established Czech consumers’ dependence on third-party certificates; Hunter (2024) underlined the role and meaning of ethical labels in five countries.

Security concerns research indicates that similar presence of national variations: D’Adamo et al. (2021) defined countries depending on their sensitivity to cybersecurity; Cardona et al. (2015) linked low institutional confidence with greater hesitance to offer personal information; Nyshadham and Ugbaja (2006) proposed differences in risky behavior meaning across cultures; Pavlou and Gefen (2004) identified institutional assurances as determinants of online trust; Jiang (2025) proved that Scandinavian consumers showed greater acceptance of computerized digital systems.

Time gains studies capture perception of time gains in various contexts: Q. Zhu and Semeijn (2015) observed saving time as a driver of behavior in the UK and the Netherlands; An et al. (2016) observed differences in motivation between nations; Devi and Kumari (2015) observed infrastructure and familiarity as mediators; Giménez-Nadal and Molina (2015) provided national disparities in time use with a context; Kyureghian and Caillavet (2025) demonstrated that time-saving facilities were valued by some demographic segments in France.

Availability and quality of information research indicates country-to-country variation in perceptions of usefulness and reliability of product information: W. Zhu et al. (2021) and Macready et al. (2025) proved differences between consumers’ interpretation of product data; Pawełoszek and Bajdor (2020) reported that experience creates trust in information; Cardona et al. (2015) assigned unclear product information to reduced cross-border activity; Mahmood et al. (2014) and Daiya (2023) highlighted how market maturity and social trust affect information trustworthiness; Gracz (2024) highlighted transparency and user-friendliness in Poland; Stoklasa and Halaška (2025) highlighted the relevance of quality labels in the Czech Republic. Shopping services satisfaction studies maintain that the way consumers report their satisfaction is a result of infrastructural, psychological, and cultural determinants: Kemper (2018) proved that individualism and power distance cultural determinants influence perceived service quality among six European countries; Kristensen and Johansson (2008) proved that response patterns differ across the various regions of Europe even with similar conditions, with Nordic customers reporting higher satisfaction; Pedersen and Schmidt (2011) linked satisfaction to digital ecosystems viability, with an emphasis on national readiness.

Based on the scientific findings of the literature review, all the seven analyzed di-mensions result in national differences. The literature consists mainly of studies in a single country or for one region. Research focuses on a single specific dimension of consumer behavior, although in most cases focuses on sub-dimensions.

This can be interpreted as a research gap. All the dimensions being researched across a sample of selected countries are considered in this paper. Therefore, multidimensional and multinational context is attained, which the literature does not explore. Contributing to the existing knowledge also helps to place the differences in online consumer behavior in the European market.

## 3. Methodology

To test the hypotheses concerning differences in factors influencing online consumer behavior across samples of selected European countries, research was conducted. Primary data were collected through a questionnaire-based survey. For each hypothesis, specific questions were designed to capture variables corresponding to the respective analytical dimensions discussed in the literature review. All survey items (Table 1) were phrased in a neutral and accessible manner to ensure clarity for respondents. Responses were measured using a 5-point Likert scale where: 1—Strongly agree, 2—Agree, 3—Neither agree nor disagree, 4—Disagree, 5—Strongly disagree.

This study focuses on a selected sample of countries: Spain, France, Poland, and Russia. This sample represents a diverse structure of consumers across Europe, covering both western and eastern regions of Europe (Table 2). Spain, France, Poland and Russia together with their population represent a broad spectrum of consumers who are diverse in terms of institutional, cultural and economic conditions in Europe. Similar combinations of countries in consumer behavior studies were used in the research of (Piekut & Knapková, 2025; Stávková & Sharma, 2005; Pellandini-Simányi & Gulyás, 2018).

The sample was obtained using non-probability convenience sampling, primarily through online distribution via social media platforms. While this approach allows efficient access to active online shoppers, it may introduce self-selection bias and limit representativeness of general national populations. Nevertheless, the obtained sample reflects demographic profiles typically associated with frequent online shopping behavior, as younger and higher-educated consumers are generally more active in digital purchasing environments (Eurostat, 2024; Ecommerce Europe, 2024). The questionnaire is provided in Appendix A. A pre-test was conducted with 10 respondents from each country prior to the main data collection. No issues were reported during this phase, and no missing data was identified in the dataset during the data collection and data processing phases. As a result, no imputation or exclusions of data were performed.

Demographic characteristics of respondents from the four countries were analyzed with respect to gender, generational cohort, education level, online shopping preferences, and expected delivery times (Table 1):

- In Spain, 154 respondents participated. Men represented 59.1% of the sample, which differed from the other countries. The largest group was Generation Z (53.9%), followed by Generation Y (30.5%). Most respondents had completed first-level tertiary education. Free shipping was the most common preference, and the most frequently expected delivery time was 4–7 days.
- In France, 158 respondents took part, with women comprising 65.8% of the sample. Generation X represented 44.3%, followed by Generation Z (47.5%), while Generation Y accounted for a smaller share. Most respondents had attained second-level tertiary education. The expected delivery time was again most commonly 4–7 days, and preferences were divided between free delivery and reduced shipping costs.
- In Poland, 300 respondents participated. Women represented 70.7% of the sample. Generation Z accounted for 86.3%, followed by Generation Y (13.0%), with Generation X being a small minority. The highest proportion of respondents completed secondary education, followed by first-level tertiary education. Free or reduced-cost delivery was most preferred, with 4–7 days being the most frequently expected delivery time.
- In Russia, 151 respondents participated, with women comprising 62.9% of the sample. Generation Z represented 68.2%, followed by Generation Y (23.2%) and Generation X (8.6%). Most respondents had completed first-level tertiary education, followed by second-level tertiary degrees. The majority preferred delivery within 4–7 days, and either reduced-cost or free shipping options were most frequently selected.

The data analysis itself consisted of several steps. Assessing internal consistency was the first step in data analysis. In this step, it was necessary to determine whether the values of the responses for survey items (26 in total; Table 1) within each dimension can be considered as internally consistent. If consistency is present, aggregation into composite dimension-scores is suitable.

Composite score as arithmetic means was calculated for each dimension and related survey items. This composite score was used as a dependent variable in subsequent tests. Aggregation of individual items into composite scores requires internal consistency, meaning reflection of the same underlying construct. Cronbach’s alpha is metric that is commonly used to measure such consistency. Survey item groupings into dimensions were based on conceptual and thematic coherence and not on psychometric validation. Survey items within each dimension capture more than one psychological aspect. For this reason, this metric was not appropriate and therefore not used. In other words: use of Cronbach’s alpha could lead to misleading results.

Average inter-item correlation (AIIC) was used as an alternative that serves as descriptive consistency metric. As Briggs and Cheek (1986) recommend: AIIC is a suitable metric for small items sets and can be considered as an appropriate metric when in survey item sets thematic consistency is present. AIIC represents the mean of all Pearson survey item correlations for each dimension. AIIC values of 0.15 and 0.50 are interpreted as acceptable internal consistency.

It should be noted that no exploration of confirmatory factor analysis was conducted. Our research objective was not to develop or validate a psychometric scale of dimensions survey items. Dimensions were constructed on the basis of conceptual and thematic coherence that is in line with practices used in exploratory research. As Churchill (1979) and Gerbing and Anderson (1988) noted, theoretically grounded dimensions can serve as analytically meaningful units in exploratory studies, even if psychometric unidimensionality is not initially confirmed.

AIIC measurements were calculated separately for each country (Table 3). Several dimensions showed acceptable or strong internal consistency. Dimensions B (Price sensitivity and perceived cost advantages) and G (shopping service satisfaction) measured 0.5 for 3 out of 4 countries. Not all dimensions met criteria and did not show strong consistency. Dimensions A (shipping-related concerns) and E (time-related benefits) measured 0.5 for only 1 out of 4 countries. Country-perspective (e.g., Poland) AIIC showed that in some cases values fell even below 0.15, indicating not acceptable internal consistency.

Excessively high internal consistency indicated by high AIIC values (above 0.5) may signal redundancy of survey items within a single dimension. Redundancy may reduce dimensional breadth by inclusion of items that are too similar. This may lead to limiting of the scale’s ability to distinguish between respondents.

Although the internal consistency analysis revealed weakness in internal consistency of certain dimensions and countries, these weaknesses were considered acceptable for country and dimension comparisons in an exploratory context. Even if weak internal consistency was observed, the questionnaire was not designed to explore patterns in consumer perceptions across conceptually and thematically related topics and, as already mentioned, notation from Churchill (1979), Gerbing and Anderson (1988) indicates that theoretically grounded dimensions can serve as analytically meaningful units in exploratory studies, even if psychometric unidimensionality is not initially confirmed. Composite scores of dimensions were retained, and interpretation of weakness was considered in further analysis.

The second step in data analysis was to assess the normality of the data distribution. The Shapiro–Wilk test was applied to the variables derived from survey items. Each dimension was operationalized as a composite scale calculated by averaging responses to its corresponding survey items, reflecting the underlying construct being measured.

In each case (Table 4), the *p*-value was well below 0.05, indicating deviation from normality. All *p*-values were below 0.0001, suggesting that the distributions of responses for each dimension did not meet the assumption of normality.

Although the violation of normality may be less critical with large sample sizes due to the central limit theorem, the ordinal nature of the Likert-scale data and the need to verify variance assumptions warranted further testing. Therefore, Levene’s test was conducted for each dimension to assess the homogeneity of variances (Table 5). Results of test guided selection of suitable ANOVA method. In terms of limitations, it should be further noted that low internal consistency (Table 3) in some dimensions and countries may contribute to the heterogeneity of variances across dimensions.

Based on the results of Levene’s test (Table 4), the choice of ANOVA procedure was adjusted according to established guidelines:

- If Levene’s test indicated equal variances (*p* ≥ 0.05), one-way ANOVA was applied. Although the assumption of normality was violated, large sample sizes (n > 150 per country) allow the use of ANOVA under the central limit theorem. The assumption of homogeneity of variances remained necessary for this procedure.
- If Levene’s test indicated unequal variances (*p* < 0.05), Welch’s ANOVA was used. This procedure accounts for heterogeneity of variances and is appropriate when group variances are unequal.

Following statistically significant results from ANOVA, post hoc tests were conducted depending on established guidelines on which ANOVA variant should be applied:

- For one-way ANOVA, the Tukey HSD test was used to evaluate pairwise group differences. Tukey HSD controls the family-wise error rate under the assumption of equal variances.
- For Welch’s ANOVA, the Games–Howell test was used. This test does not require equal variances or equal sample sizes and controls Type I error without assuming homogeneity of variances.

All statistical analyses were conducted at a significance level of α = 0.05.

One-way ANOVA and Welch’s ANOVA both give the *p*-value as output.

When ANOVA and post-hoc tests’ *p*-value is less than the chosen significance level (*p* < 0.05), statistical significance is obtained. It is interpreted to mean that observed differences are unlikely to be the result of random chance, and the null hypothesis of no difference between group means is rejected. If the *p*-value is greater than or equal to the significance level (*p* ≥ 0.05), statistical significance is not reached, and differences are estimated to most probably be due to random variation.

Statistical significance alone is meant to suggest the possibility of the existence of data difference at the cost of practical significance or size. With respect to this, size measures were employed:

- Eta squared (η^2^) was used for one-way ANOVA. It indicates the proportion of total variance on the dependent variable that is explained by the independent variable (group differences). The eta squared scale is from 0 to 1; larger values indicate a larger proportion of variance explained by the factor.
- η^2^ is not appropriate for Welch’s ANOVA because its calculation relies on the equal variances’ assumption between groups. Since Welch’s ANOVA is performed under the condition of this assumption being violated (i.e., variances are unequal), calculation of eta squared will provide a biased and possibly distorted effect size estimate. Omega squared (ω^2^) was employed with Welch’s ANOVA. ω^2^ gives a less biased population estimate of effect size with a more accurate reflection of the factor’s true contribution, particularly with heterogeneous variances.
- For the pairwise comparisons made using Tukey’s HSD (following one-way ANOVA with homogeneous variances), Cohen’s d was used. Cohen’s d is an appropriate statistic because it relies on a pooled standard deviation, which is valid under the assumption of equal variances.
- Conversely, for pairwise comparisons from the Games–Howell test (following Welch’s ANOVA with heterogeneous variances), Hedges’ g was utilized. Hedges’ g is preferred over Cohen’s d in these scenarios, as it includes a correction for small sample sizes and is more robust when variances are unequal or sample sizes differ, aligning with the very reasons for using Welch’s ANOVA and Games–Howell.

For η^2^ or ω^2^, effect size values of around 0.01 were considered as small, 0.06 as medium, and as large around 0.14. Similarly, if Cohen’s d or Hedges’ g was used, an effect size of around 0.2 was considered as small, around 0.5 as medium, and around 0.8 as large.

A clearer understanding of the results is provided when a statistically significant *p*-value is paired with a meaningful effect size. The *p*-value implies that the difference that was found is likely, whereas the effect size is the indication of the practical importance of the difference. They allow us to assess both the occurrence of a difference and the practical significance that this difference implies.

During the course of analysis, the authors applied the use of ChatGPT (GPT-4 model) in helping provide Python (version 3.11) scripts (using packages like pandas, SciPy, Statsmodels, Pingouin, and scikit-posthocs). The scripts were applied in the processing of data as well as in statistical analyzes, some of the statistical analyzes being average inter-item correlation (AIIC) in measuring internal consistency, the Shapiro–Wilk test in checking normality, Levene’s test in checking homogeneity of variances, bar chart with error bars (95% confidence interval) as the alternative to the use of ANOVA, one-way ANOVA, Welch’s ANOVA, post hoc measures like Tukey’s HSD test and Games–Howell test, eta squared and omega squared and Cohen’s d and Hedges’ g in measuring the effect size. The selection of statistical procedures, the implementation of the code, the control of the calculations as well as the verification of the results were independently performed, supervised, and confirmed by the authors.

## 4. Results

**Figure 1:**
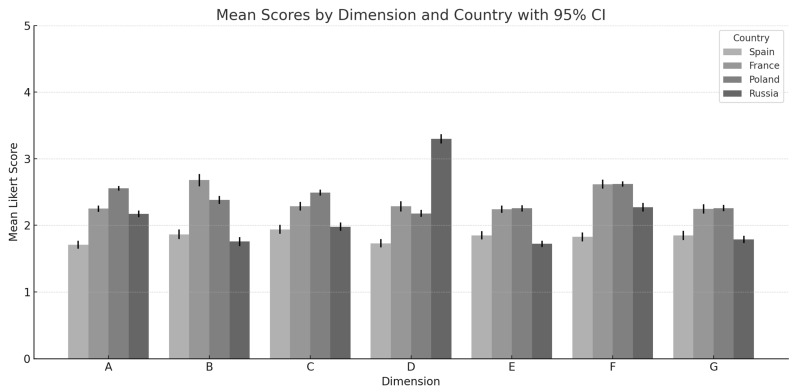
Mean Likert-scale scores across dimensions (A–G) by country with 95% CI.

Presenting the summary of differences in consumer opinions, Figure 1 shows a grouped bar chart with the average Likert-scale rating (on a scale from 1 to 5) per dimension (A–G), by country. Error bars for standard errors of the mean, approximately 95% confidence, are displayed on the chart.

The graph reveals that there are some dimensions with significant differences between countries, particularly in dimension D and even more in dimension G. Low national differences in some dimensions are revealed in the average scores, which reflect similar consumer views in different countries.

The cross-country and dimension comparison required Levene’s test to investigate homogeneity of variances. The comparison utilized Welch’s ANOVA on six out of seven dimensions (A–G) as unequal variances encountered. Standard one-way ANOVA was used for dimension C as equal variances were determined. The results of Levene’s test are presented in Table 6.

For all seven dimensions, country differences were significant at the *p* < 0.05 level, indicating cross-national variation in the studied behavioral domains. For further evaluation of the practical significance of the differences, effect sizes were calculated and can be seen in Table 7.

Findings demonstrate difference in perception of consumers across countries. Dimensions A, B, and D are present in the form of high effect sizes, reflecting large and practical differences in thinking or feeling between countries. Conversely dimensions C, E, F, and G were reasonably different, not excessively, but quite a lot.

These patterns were further examined with post hoc tests that seek to identify differences between pairs of nations on each dimension. These post hoc analyses better specify the sources of these differences and provide additional richness to the interpretation of findings.

Statistical significance was observed for all dimensions investigated in validating cross-national differences. This does not by itself determine if these differences are of practical significance. To augment the pair-wise tests’ statistical significance, effect sizes were calculated: Hedges’g was applied on Games-Howell tests (for Dimensions A, B, D, E, F, and G), while Cohen’s d was applied on the Tukey HSD test (for Dimension C). It should be noted that negative effect sizes (as presented in Table 8) are to be interpreted as indicating an inverse relationship or reduction, depending on the variables involved.

Dimension A: Statistically significant differences existed in five out of six country pairings. Of the two large effect size ones, the France–Russia and Poland–Russia pairings were large, and the France–Poland and Poland–Spain pairings were medium-sized. This indicates that the difference between the countries is not merely statistically significant but also sufficiently sized in real terms. Conversely, France to Spain comparison revealed a negligible, non-significant effect and indicated that the two Western European countries’ customers share the same perceptions of shipping. Generally, this factor identifies differences between nations, and that is particularly evident in differences between the customers in the East and the West of Europe.

Dimension B: Statistically significant differences were obtained in five out of the six country comparisons. Two of them, the France–Russia and the France–Spain pairings, had large effect sizes, and the France–Poland, Poland–Russia, and Poland–Spain pairings had medium-sized effects. The effect is that the difference in the price sensitivity as well as the perceived cost benefit between the countries is not only statistically significant but also of practical significance—especially when the comparison is performed using France versus Russia or Spain. The only comparison that was not significant was the use of the Russia and Spain comparison, which also had a small effect. The inference is that the consumers from the two countries have the same thinking as far as prices are concerned.

Dimension C: Five of the six countries’ comparisons were statistically significantly different from one another and had medium effects for all. The same general trend here would mean that citizens of most countries have moderate but still measurable levels of product quality. The only exception was the Spain–Russia comparison, which was nonsignificant and reflected only a small effect. The latter reflects hardly any difference in the assessment of quality between the two countries.

Dimension D: Five out of six country comparisons showed significant differences. Three of them, i.e., France–Spain, Poland–Spain, and Russia–Spain had large effect sizes, i.e., strong and significant differences in transaction security perception. The other two significant comparisons, i.e., France–Russia and Poland–Russia also contained medium effect sizes, i.e., noticeable differences. Only the comparison between France–Poland did not prove to be significant. It also had a small effect size, i.e., respondents from both countries have the same view overall in the field of online security.

Dimension E: Four out of six cross-country comparisons were significant. The French–Spanish comparison had the greatest effect size, i.e., there is an obvious and a large difference in the observation of time-related benefits. The remaining three comparisons, that is, France–Russia, Poland–Russia, and Poland–Spain yielded medium effect sizes, thus signifying moderately significant differences. The comparison of Russia and Spain yielded a statistical significance, but the effect size was small, thus the difference in practice is again likely to be trivial. The comparison of France and Poland yielded no significant difference either statistically or in practice.

Dimension F: Statistically significant differences were present for five of the six country comparisons. Two of them, France–Russia and Poland–Russia, possessed large effect sizes, i.e., significant differences in how much people trust information that is presented on sites. The other three of the comparisons, that is, France–Spain, Poland–Spain, and Russia–Spain, were of medium effect sizes, meaning that the differences both statistically and practically were significant. The France–Poland comparison was not significant and was of small effect only, thereby the implication is that the two nations’ consumers are similar in such a way.

Dimension G: Four of the six countries’ comparisons were statistically significant, all with medium effect sizes. These results indicate that although the differences were uniform, they were only of practical, medium size. Two of the comparisons, France–Poland and Russia–Spain, were small in effect size. Of these, only France–Poland was statistically significant, implying that there was a real but small difference.

The outcomes reflect that the widest variations between nations appeared in three wide dimensions: security (Dimension D), shipping concerns (A), and price sensitivity (B). Russia and the Western European countries often showed medium to high effect sizes in these dimensions, which reflected clear disparity in consumers’ perception in the above areas. These discrepancies likely stem from more broadly based institutional as well as infrastructure-related issues like varying degrees of confidence in Internet payment platforms, dependability in the logistics field, or price expectations that can be established at the national income as well as marketplace levels. Russian and Polish consumers were more preoccupied with respect to reliability of delivery as well as security in the purchase in comparison to the Western European consumers. French consumers were unique in exhibiting other price sensitivity patterns both in comparison with the respondents of the East as well as the West of Europe.

The least significant differences were discovered regarding service satisfaction of shopping in Dimension G. Although statistical significance was discovered for some differences, small to medium effect sizes were obtained. This suggests that online service experience is increasingly becoming standardized across consumers in Europe. Worldwide standardization of site appearance, successful deliveries, and the spillover from the world’s leading e-commerce titans are likely forces behind this development. The same pattern was realized in Dimension C, perceived product quality, with national variation being moderate. This is a testament to universal standard expectation in products, propelled through increased global brand growth and universal protection laws across the European marketplace.

The most similar pair across dimensions was Poland and France. Along dimensions such as availability of information (Dimension F) and security of transaction (Dimension D), differences between the two were zero or close to zero (statistically) or with near-zero effect sizes. The similarity can be attributed to similar regulation frameworks under the policies of the EU digital market prevailing, despite the contrasting consumer cultures prevailing in the two nations.

The research discovers there is noticeable cleavage along the East vs. West Europe dimension in how consumers define risk vs. reliability. At the same time, agreement is rising in nations on the question of product quality and platform-oriented services.

Table 9 disaggregates it. Across all the dimensions, there were over 67% of the pair-wise comparisons of nations that had statistically significant differences, indicating that national setting always affects the way consumers think about online shopping. Statistical results, though, are just half the tale—most of these differences also appear substantively significant in the actual world.

Most comparisons were above the level of practical significance, i.e., at least medium or large effect size. With Dimensions A, B, C, D, and F, more than 17% did so at this level, but Dimensions E and G were even more impressive in the sense that more than 33% of the differences at this level were practically significant. These findings thus indicate that although the magnitude of difference varies along the dimension, the online shopping perception of the consumers varies between countries.

The comparisons validate all the hypotheses that have been put forward. Each of these (H(A)–H(G)), indeed, was vindicated with significant inter-country differences being detected along all the behavioral dimensions under examination. These findings continuously substantiate the hypothesis that consumer online shopping perceptions differ along national lines (Table 10).

## 5. Discussion

### 5.1. Summarization

The study verifies that consumption behavior in the online realm differs significantly across countries, with all measures being significantly dissimilar from one another: with strong support for hypotheses H(A) through H(G). Nevertheless, all regions were not affected similarly. The magnitude of variation varies from dimension to dimension, gaining an understanding of what aspects of online retailing are driven by national environment and culture.

Among the most pronounced differences was price sensitivity and perceived cost advantage (Dimension B), where five out of six pairwise comparisons between countries reached statistical significance. This suggests that consumers’ perceptions of pricing benefits in e-commerce are highly context-dependent—shaped by factors such as national purchasing power, cultural norms regarding price fairness, and institutional trust in price transparency (Baye et al., 2002, 2007; Cardona et al., 2015; Hinz et al., 2011; Kemper, 2018; Kristensen & Johansson, 2008; D’Adamo et al., 2021; Gracz, 2024; Sendcloud, 2022).

The same was the case with shipping-related aspects (Dimension A), which also showed remarkable national variations. These findings agree with previous research on unbalanced logistics performance, consistency of deliveries, and fulfillment expectations in Europe (Vasić et al., 2021; Majchrzak-Lepczyk, 2019; Grant & Philipp, 2014; Kim et al., 2017; WIK-Consult, 2021; Pavlou & Gefen, 2004; Stoklasa & Halaška, 2025; Hunter, 2024). These differences are most likely due to national variations in infrastructure and the degree that consumers place trust in the deliverers and services providers.

The same level of variation was found in security concerns (Dimension D) and quality perception (Dimension C). For both dimensions, the variation at the country level is likely the result of institutional trust, experience with online information protection or information fraud in the past, and expectations with regard to product information precision and the authenticity of measures of quality (Pavlou & Gefen, 2004; D’Adamo et al., 2021; Jiang, 2025; Știr, 2018; Pawełoszek & Bajdor, 2020; Pedersen & Schmidt, 2011; Hunter, 2024). The two dimensions seem to mirror the overall confidence that customers have in the national institutions as well as digital infrastructures, as shaped through the repetitive exposure that they experience with online services.

Significant differences also emerged in the dimension of information availability and quality (Dimension F). These findings agree with previous research findings of variation in digital literacy, transparency of the platforms, accessibility of the data, and institutional credibility in countries (W. Zhu et al., 2021; Macready et al., 2025; Daiya, 2023; Pawełoszek & Bajdor, 2020; Grant & Philipp, 2014; Kim et al., 2017; Huterska & Huterski, 2022). Perceptions of information seem as much shaped by technical considerations as broader-based trust in the governance of data and the responsiveness of digital platforms.

In contrast to the highly differentiated dimensions discussed above, time-related benefits (Dimension E) and overall satisfaction with shopping services (Dimension G) showed comparatively less variation across countries. Despite some statistically significant differences that arose, the overall patterns reveal some convergence in the perception of time efficiency and satisfaction with services. The relative convergence is perhaps in part the result of similar expectations regarding usability and convenience in online shopping that become as consistent as possible through universal design of platforms and general standards of services (Q. Zhu & Semeijn, 2015; An et al., 2016; Devi & Kumari, 2015; Kyureghian & Caillavet, 2025; Baye et al., 2002; Kemper, 2018; Pedersen & Schmidt, 2011). However, the perception is in part mediated through cultural dispositions toward customer services, response style in questionnaires, and perceived justice in the experience of services (Kemper, 2018; Kristensen & Johansson, 2008; Hunter, 2024; Vasić et al., 2019).

The findings reflect that there is a multilevel structure of deviations across countries. Variables such as price sensitivity, shipment, security, perceived quality, and availability of information are more contextually sensitive as they are shaped by institutional trust, economic profile, infrastructural maturity, and cultural beliefs. However, time efficiency and satisfaction reflect more globally consistent standards but are moderated through local norms.

Overall, then, what these findings reveal should be tempered in terms of the quality of the measurement. Individual variation in internal consistency, as reflected in the patterns across dimensions and countries in the AIIC values, was almost certainly responsible for the variation across countries. Such appreciation underlines the importance of cautious interpretation and recommends the future work that is needed to validate and refine measures of consumption behavior online.

### 5.2. Theoretical Implications

In addition to the empirical evidence that extends our knowledge of online consumer behavior, some theoretical contributions can be presented as below:

The comparison revealed significant and considerable statistical differences across countries in all seven aspects of online shopping behavior, thus providing strong support toward hypotheses H(A) through H(G). Of the specific comparisons across the country pairs, specifically, there were 34 out of 42 (81%) that were significant in the post-hoc comparisons. These results support that national environmental factors significantly contribute to consumer perception, validating previous theories explicating the importance of national institutions (Pavlou & Gefen, 2004; Gautam, 2024; Walczuch & Lundgren, 2004; Zhao et al., 2022) and cultural norms (Pratesi et al., 2021; Shi, 2023; Zimu, 2023) in shaping digital consumption behavior.

The research provides a systematically structured model in the examination of online consumption behavior in its decomposition into seven independent conceptual dimensions. The model enables notable cross-country comparisons based on composite indicators. Its effective application with the sample of 763 respondents remains strong even in the event of departure from normal distribution or equal variances of the datasets. However, the internal consistency of certain composite dimensions varied across countries as indicated by the existence of variation in average inter-item correlation (AIIC) values. Such inconsistency is liable to affect the effect size or stability of some observed dissimilarity, and future research is therefore particularly important in psychometrically validating and refining the dimensions in culturally diverse application contexts.

The findings that 84% of comparisons in Dimensions A (shipping-related matters), B (price consciousness), C (perception of quality), D (security-related issues), and F (availability and information quality) demonstrated significant variation indicate that, despite shared patterns of technology and governance, national variations in the manner that citizens act as consumers continue. The evidence supports local context-based theories (Chan, 2018; Di & Peng, 2017; Dudziak et al., 2023) and contradicts the hypothesis that generic patterns of consumer behavior can adequately account for the manner that individuals interact with digital retail in various nations (Nevo, 2010; Rau & Samiee, 1981; Tesic & Bogetić, 2022).

Some of the differences found in this study can be explained with well-known theories of psychology and culture. For example, the more significant security issues (Dimension D) that were present in some countries may reflect more avoidance of uncertainty, as theorized in Hofstede’s cultural dimension framework (Taras et al., 2010). Again, quality perception differences (Dimension C) and differences in prices (Dimension B) may relate to risk aversion and the online trust formation processes (Pavlou & Gefen, 2004). Though this study did not test such underlying processes directly, findings align with theorized accounts connecting national environment with the behavior of consumers through more general social structures and psychological dispositions.

### 5.3. Practical Implications

Results of this study may inform commercial strategies and policy approaches in digital commerce. Implications arising from the identified cross-national variations include the necessity of a differentiated market-aware strategy for Europe-based e-commerce firms. The generic strategies rarely succeed as they attempt to fulfill the specific expectations, concerns, and sensibilities of national-level consumers. If online stores with an orientation toward Europe want to succeed in the diverse markets of Europe, generic strategies will no longer suffice. Businesses that do business online should use localized strategies that consider how people in each country act and how they see risk. In short, cross-border e-commerce will only work if people know a lot about the cultural norms, how much they trust institutions, and how well the local infrastructure is developing. These variations frequently emanate from wider cultural and psychological characteristics—such as the extent of acceptable uncertainty, response to risk, as well as ease in building confidence. That is the reason shopping behaviors may significantly differ from one nation to the next, including among countries that may seem similar at the surface level.

These findings offer practical pointers for the people working in cross-border e-commerce—companies, platform teams, and regulators. It should be noted that measurement reliability was not even. In some areas reliability differences were observed.

Given that 84% of country comparisons on Dimension B show meaningful differences, a uniform pricing playbook is unrealistic. Businesses should adjust the way prices are shown, when promotions happen, and the value cues that are most important to fit with local ideas of fairness. Poland is a country where people are very sensitive to prices, so clear prices, clear discounts, and simple loyalty rewards are likely to be popular. In France, by contrast, greater weight is placed on service quality and brand credibility; heavy discounting is often less effective—and is, at times, counterproductive.

The differences in consumer concerns around shipping and logistics (Dimension A), with 84% of country comparisons showing significant variation, highlight the need for fulfillment services to reflect local expectations. In places like Russia or Poland, where people may be more cautious about delivery reliability or return policies, these concerns could stem from lower trust in institutions or a greater sensitivity to uncertainty. Businesses that work in these kinds of markets should think about ways to build trust, like offering real-time tracking, weekend delivery, or being more open about what happens after a purchase. These kinds of personalized strategies can help build trust with customers, make them happier, and lower the chances that they will leave their carts.

Differences on Dimension D, which gauges how safe online shopping feels and how reliable information is perceived, highlight the critical role that trust plays in e-commerce. Unless consumers have faith in a platform’s security measures, purchases are more likely to stall in markets with lower institutional trust (like Russia). Strong payment protections, independent security badges, and easily readable privacy policies are the best ways to demonstrate assurance and build trust. Implementation alone, however, is insufficient; the signals must be obvious and not obscured. They tend to decrease hesitation when placed prominently (pre-checkout, in the cart, at sign-in), especially among risk-averse customers who value safety over convenience or speed.

There are also clear differences between countries in how people feel about service quality (Dimension C) and overall satisfaction (Dimension G). To close these gaps, more than just changes in business will be needed; policy support is also important. Raising the bar can be accomplished by making people more digitally literate, making institutions more open, and putting money into better logistics systems. These steps are very important in Central and Eastern Europe, where e-commerce is still growing and people are still learning to trust it.

## 6. Conclusions

This study aimed to examine whether consumer perceptions related to online shopping behavior differ across European countries, by testing cross-national differences across seven behavioral dimensions. The analysis identified variation in consumer perceptions related to shipping-related concerns and preferences, price sensitivity and perceived cost advantage, quality perception, security concerns, time-related benefits, availability and quality of information, and shopping service satisfaction across four countries: Spain, France, Poland, and Russia. The empirical analysis confirmed statistically significant differences across all dimensions, providing robust support for the proposed hypotheses H(A) through H(G).

There are some limitations worth mentioning. While the rather large sample sizes did allow the use of parametric statistical analyses, it should be remembered that Likert-scale data is ordinal, and this imposes some limits on analysis. Internal consistency of composite dimensions, measured in terms of average inter-item correlation (AIIC), also varied between countries. In some cases, especially for such dimensions as shipping problems and time benefits, internal consistency was low, which would affect findings on how stable and credible the results would be. On the other hand, high AIICs in some dimensions would imply that items were too close to one another, reducing the ability to measure a more general construct or distinguish between different aspects of it. Yet another significant limitation is that the structure of the measurement tool wasn’t piloted using exploratory or confirmatory factor analysis (EFA or CFA). Without that, it becomes harder to say whether the dimensions are really measuring what is supposed to be measured or if they are really equivalent across cultures. Because the item clusters were thematic in design and not psychometrically validated, this investigation should be viewed as exploratory—a precursor to more research of greater intensity.

The four countries chosen were the regional focus, which restricts generalizability to broader settings. Unequally sized numbers in the sample and convenience sampling could limit representativeness. The sample gathered may not effectively reflect national population make-up in terms of age, gender, or socioeconomic status, possibly introducing sample bias. Sample composition is an important factor. In particular, the Russian and Polish subsamples had higher representation of young, female, and digitally active respondents. This might have amplified perceived security concern differences, information quality, or consumer tastes. Thus, some of the cross-national differences that were identified in the research can be accounted for more on demographic grounds than real national-level differences. The assessments rely on self-reporting, which can be subject to biases such as social desirability effects, recall lapses, and individual interpretation of survey items. The cross-sectional design provides a snapshot of perception at a single time point without controlling for potential temporal processes. The analysis was limited to selected dimensions of consumer conduct in online shopping, primarily the product, platform, and delivery properties, while other variables such as social interactions, psychological attributes, or macroeconomic conditions were excluded. The findings should be interpreted in the context of countries investigated and cannot always be applied to other European or global markets with dissimilar cultural, institutional, or economic features.

Geopolitical developments should also be considered when interpreting the results related to Russia. Data collection was conducted prior to the escalation of the military conflict and heightened geopolitical tensions involving Russia. These subsequent developments may have influenced consumer attitudes and trust in e-commerce services. As such, caution is advised when generalizing the findings for Russia to current or future conditions.

Future research can address several of these shortcomings. Extension of the geographic scope to include additional European and non-European countries would allow for broader cross-regional comparisons. Utilization of representative or stratified sampling frames would increase generalizability and better reflect national population profiles. In this study, the use of convenience sampling led to overrepresentation of young, female, and digitally literate respondents—especially in certain country samples like Poland and Russia. This demographic bias may have influenced the results in areas such as security concerns, information quality, and delivery expectations, and possibly reflects trends linked to age or gender rather than real national difference. To carry these results further, future research needs to achieve more representative samples and, furthermore, explore how demographic variables interact with different aspects of online consumer behavior. Longitudinal research designs (i.e., repeated observations of the same or similar respondents over time) would enable the analysis of how consumer perceptions evolve as digital infrastructures develop, and regulatory frameworks adapt over time. Incorporating additional behavioral dimensions, including social dynamics, psychological characteristics, and macroeconomic indicators, may offer a more comprehensive understanding of online shopping behavior across different markets. Although the questionnaire was carefully designed to minimize common response biases, self-reported survey data may still be subject to potential limitations such as subjective interpretation or individual response styles. To further strengthen validity, future studies may complement survey data with behavioral transaction records or observational data sources. The use of mixed-method approaches, including qualitative interviews, may further enrich the understanding of cultural and psychological mechanisms underlying observed cross-national differences.

Future research could also be enabled using innovative statistical methods to explore interactions between the different dimensions. For example, regression-based analyses, including moderation or mediation analysis (e.g., PROCESS modeling), can be utilized to establish whether variables like perceived product quality or security concerns are involved in mediating the relationship between price sensitivity and customer satisfaction, or whether the relationships vary by national setting. Deploying such models would yield a more nuanced insight into how different dimensions of consumer perception work together and impact online shopping behavior. This, in turn, would increase the explanatory power and richness of future research on this topic. Such research could yield findings that are useful for commercial purposes as well as policymakers who are keen to foster trust, improve service quality, and support sustainable development of cross-border digital trade.

Overall, consumer behavior in the online context cannot be conceived as an undifferentiated phenomenon. Instead, it is shaped by a set of interrelated cultural, institutional, and economic factors that vary from country to country. To be effective in e-business, business and research strategies must take such national differences into consideration. Doing so avoids oversimplifying assumptions and promotes more accurate, responsive, and inclusive strategies that capture effectively differentiated realities of global consumers.

## Figures and Tables

**Table 1:** Dimensions and survey items.

Dimension	Survey Item
A: Shipping-related concerns and preferences	Free shipping makes me more likely to buy something online.
	I worry that my online order might not be delivered.
	I worry that I might get the wrong product when I shop online.
	I worry that clothes I order online might not fit.
	I worry that products I order online might not be fresh or good quality.
	I can choose weekend delivery when I shop online.
B: Price sensitivity and perceived cost advantage	I think online shopping helps me save money.
	I spend less money per purchase online than in stores.
	I spend less per order when I shop online compared to in stores.
C: Quality perception	I think the quality of products I buy online is similar to what I buy in stores.
	I get similar buying conditions online as I do in physical stores.
	The products I order online usually match what I see on the website.
D: Security concerns	I feel uncomfortable entering my card details when shopping online.
	I am concerned about losing my privacy when I shop online.
	I worry that my identity could be stolen when I shop online.
E: Time-related benefits	I save time when I shop online.
	I like that I can shop online at any time.
	Online shopping is a good way for me to spend my time.
F: Availability and quality of information	The product information I see online is the same as what I find in stores.
	The product information I see online is clear and accurate.
	The product information I see online is up to date.
G: Shopping service satisfaction	I am happy that I can shop online.
	I find online shopping interesting.
	I would recommend online shopping to others.
	I enjoy buying things online.
	I think online shopping is a good way to shop.

**Table 2:** Sample demographics.

Demographics		Spain	France	Poland	Russia
Gender	Total	154	158	300	151
	Female	63	104	212	95
	Male	91	54	88	56
Age	Generation X	24	70	2	13
	Generation Y	47	13	39	35
	Generation Z	83	75	259	103
Education level	Primary education	0	0	61	0
	Secondary education	12	15	138	18
	Tertiary education 1st degree	78	25	54	80
	Tertiary education 2nd degree	48	116	47	41
	Tertiary education 3rd degree	16	2	0	12
Daily time spent browsing the internet	Less than 1 h	27	19	31	4
	1–2 h	34	44	54	6
	2–3 h	33	26	59	41
	3–4 h	28	16	44	66
	More than 4 h	32	53	112	34
Approximate maximum amount spent on single online purchase	Less than 30 €	31	12	4	23
	30–50 €	48	28	46	50
	50–100 €	40	30	73	8
	100–200 €	35	88	63	65
	More than 200 €	0	0	114	5
Approximate maximum amount spent on online purchases in a year	Less than 50 €	8	5	16	7
	50–100 €	23	32	42	20
	100–200 €	48	0	94	72
	More than 200 €	75	121	148	52
Preferred delivery cost when shopping online	Prefer free shipping	106	19	62	78
	Prefer lowest total price overall	15	51	128	17
	Prefer reduced shipping cost	33	88	110	56
Estimated time of delivery	1–3 days	70	7	31	40
	4–7 days	72	90	178	105
	8–14 days	12	61	91	6

**Table 3:** Internal consistency analysis.

Dimension	Spain	France	Poland	Russia
A	0.5314	0.2074	0.144	0.3919
B	0.6829	0.6228	0.4773	0.7757
C	0.5997	0.3964	0.1838	0.592
D	0.597	0.6122	0.5426	0.5037
E	0.3569	0.2186	0.0887	0.3014
F	0.657	0.4275	0.188	0.5855
G	0.6239	0.5937	0.3169	0.5646

**Table 4:** Shapiro–Wilk test.

Dimension	*p*-Value	Shapiro–Wilk Normality Test Result
A	*p* < 0.0001	The assumption of normality was not met
B	*p* < 0.0001	The assumption of normality was not met
C	*p* < 0.0001	The assumption of normality was not met
D	*p* < 0.0001	The assumption of normality was not met
E	*p* < 0.0001	The assumption of normality was not met
F	*p* < 0.0001	The assumption of normality was not met
G	*p* < 0.0001	The assumption of normality was not met

**Table 5:** Levene test results.

Dimension	*p*-Value	Levene Test Result
A	0.0385	Variances unequal
B	0.0039	Variances unequal
C	0.9004	Variances equal
D	0.0468	Variances unequal
E	0.0003	Variances unequal
F	0.0180	Variances unequal
G	0.0003	Variances unequal

**Table 6:** ANOVA test results.

Dimension	ANOVA Type	*p*-Value	Significance
A	Welch’s ANOVA	<0.0001	Statistically significant
B	Welch’s ANOVA	<0.0001	Statistically significant
C	One-way ANOVA	<0.0001	Statistically significant
D	Welch’s ANOVA	<0.0001	Statistically significant
E	Welch’s ANOVA	<0.0001	Statistically significant
F	Welch’s ANOVA	<0.0001	Statistically significant
G	Welch’s ANOVA	<0.0001	Statistically significant

**Table 7:** Effect size measurements.

D	ANOVA Type	Effect Size Metric	Effect Size Value	Effect Size Interpretation
A	Welch’s ANOVA	ω^2^	0.1936	Large
B	Welch’s ANOVA	ω^2^	0.1512	Large
C	One-way ANOVA	η^2^	0.0822	Medium
D	Welch’s ANOVA	ω^2^	0.2519	Large
E	Welch’s ANOVA	ω^2^	0.0915	Medium
F	Welch’s ANOVA	ω^2^	0.137	Medium
G	Welch’s ANOVA	ω^2^	0.0648	Medium

**Table 8:** Post-hoc test results.

D	Post-Hoc Test	Group1	Group2	*p*-Value	Significance	Effect Size	Value	Interpretation
A	Games–Howell	France	Poland	<0.0001	Statistically significant	Hedges’ g	−0.5011	Medium
A	Games–Howell	France	Russia	<0.0001	Statistically significant	Hedges’ g	0.8030	Large
A	Games–Howell	France	Spain	0.2563	Not significant	Hedges’ g	0.1292	Small
A	Games–Howell	Poland	Russia	<0.0001	Statistically significant	Hedges’ g	1.2702	Large
A	Games–Howell	Poland	Spain	<0.0001	Statistically significant	Hedges’ g	0.6240	Medium
A	Games–Howell	Russia	Spain	<0.0001	Statistically significant	Hedges’ g	−0.6837	Medium
B	Games–Howell	France	Poland	0.0063	Statistically significant	Hedges’ g	0.2773	Medium
B	Games–Howell	France	Russia	<0.0001	Statistically significant	Hedges’ g	0.8005	Large
B	Games–Howell	France	Spain	<0.0001	Statistically significant	Hedges’ g	0.9230	Large
B	Games–Howell	Poland	Russia	<0.0001	Statistically significant	Hedges’ g	0.5205	Medium
B	Games–Howell	Poland	Spain	<0.0001	Statistically significant	Hedges’ g	0.6383	Medium
B	Games–Howell	Russia	Spain	0.2635	Not significant	Hedges’ g	0.1279	Small
C	Tukey HSD	France	Poland	0.0467	Statistically significant	Cohen’s d	−0.2543	Medium
C	Tukey HSD	France	Russia	0.0043	Statistically significant	Cohen’s d	0.4202	Medium
C	Tukey HSD	France	Spain	0.0008	Statistically significant	Cohen’s d	0.3795	Medium
C	Tukey HSD	Poland	Russia	<0.0001	Statistically significant	Cohen’s d	0.6901	Medium
C	Tukey HSD	Poland	Spain	<0.0001	Statistically significant	Cohen’s d	0.6549	Medium
C	Tukey HSD	Russia	Spain	0.9706	Not significant	Cohen’s d	−0.0505	Small
D	Games–Howell	France	Poland	0.2665	Not significant	Hedges’ g	0.1116	Small
D	Games–Howell	France	Russia	<0.0001	Statistically significant	Hedges’ g	0.6241	Medium
D	Games–Howell	France	Spain	<0.0001	Statistically significant	Hedges’ g	−1.0957	Large
D	Games–Howell	Poland	Russia	<0.0001	Statistically significant	Hedges’ g	0.5128	Medium
D	Games–Howell	Poland	Spain	<0.0001	Statistically significant	Hedges’ g	−1.2435	Large
D	Games–Howell	Russia	Spain	<0.0001	Statistically significant	Hedges’ g	−1.9376	Large
E	Games–Howell	France	Poland	0.8565	Not significant	Hedges’ g	−0.0168	Small
E	Games–Howell	France	Russia	<0.0001	Statistically significant	Hedges’ g	0.5444	Medium
E	Games–Howell	France	Spain	<0.0001	Statistically significant	Hedges’ g	0.8220	Large
E	Games–Howell	Poland	Russia	<0.0001	Statistically significant	Hedges’ g	0.5120	Medium
E	Games–Howell	Poland	Spain	<0.0001	Statistically significant	Hedges’ g	0.7223	Medium
E	Games–Howell	Russia	Spain	0.0982	Not significant	Hedges’ g	0.1890	Small
F	Games–Howell	France	Poland	0.9605	Not significant	Hedges’ g	−0.0052	Small
F	Games–Howell	France	Russia	<0.0001	Statistically significant	Hedges’ g	0.9262	Large
F	Games–Howell	France	Spain	0.0003	Statistically significant	Hedges’ g	0.4119	Medium
F	Games–Howell	Poland	Russia	<0.0001	Statistically significant	Hedges’ g	1.0504	Large
F	Games–Howell	Poland	Spain	<0.0001	Statistically significant	Hedges’ g	0.4696	Medium
F	Games–Howell	Russia	Spain	<0.0001	Statistically significant	Hedges’ g	−0.5368	Medium
G	Games–Howell	France	Poland	0.8899	Not significant	Hedges’ g	−0.0138	Small
G	Games–Howell	France	Russia	<0.0001	Statistically significant	Hedges’ g	0.4577	Medium
G	Games–Howell	France	Spain	<0.0001	Statistically significant	Hedges’ g	0.5899	Medium
G	Games–Howell	Poland	Russia	<0.0001	Statistically significant	Hedges’ g	0.4854	Medium
G	Games–Howell	Poland	Spain	<0.0001	Statistically significant	Hedges’ g	0.6029	Medium
G	Games–Howell	Russia	Spain	0.4922	Not significant	Hedges’ g	0.0784	Small

**Table 9:** Post hoc measurement results.

D	Non-Significant	Significant	% Significant	Small	Medium	Large	% Small	% Medium	% Large
A	1	5	84%	1	3	2	17%	50%	33%
B	1	5	84%	1	3	2	17%	50%	33%
C	1	5	84%	1	5	0	17%	83%	0%
D	1	5	84%	1	2	3	17%	33%	50%
E	2	4	67%	2	3	1	33%	50%	17%
F	1	5	84%	1	3	2	17%	50%	33%
G	2	4	67%	2	3	1	33%	50%	17%

**Table 10:** Summary of hypothesis testing.

Hypothesis	Dimension	ANOVA *p*-Value	Post-Hoc Differences	Supported
H(A)	Shipping-related concerns and preferences	<0.0001	5/6 pairs significant	Yes
H(B)	Price sensitivity and perceived cost advantage	<0.0001	5/6 pairs significant	Yes
H(C)	Quality perception	<0.0001	5/6 pairs significant	Yes
H(D)	Security concerns	<0.0001	5/6 pairs significant	Yes
H(E)	Time-related benefits	<0.0001	4/6 pairs significant	Yes
H(F)	Availability and quality of information	<0.0001	5/6 pairs significant	Yes
H(G)	Shopping service satisfaction	<0.0001	4/6 pairs significant	Yes

## Data Availability

Data will be made available on request.

## Appendix A

Country: - Spain - France - Poland - Russia Age: - 15–28 (Gen Z) - 29–44 (Gen Y) - 45–60 (Gen X) Education level: - Primary education - Secondary education - Tertiary education 1st degree - Tertiary education 2nd degree - Tertiary education 3rd degree Daily time spent browsing the internet: - Less than 1 h - 1–2 h - 2–3 h - 3–4 h - More than 4 h Approximate maximum amount spent on single online purchase: - Less than 30 € - 30–50 € - 50–100 € - 100–200 € - More than 200 € Approximate maximum amount spent on online purchases in a year: - Less than 50 € - 50–100 € - 100–200 € - More than 200 € Preferred delivery cost when shopping online: - Prefer free shipping - Prefer reduced shipping cost - Prefer lowest total price overall Estimated time of delivery - 1–3 days - 4–7 days - 8–14 days

For each of the following statements, please indicate your level of agreement by selecting the response that best reflects your opinion.

Use the following scale: 1—Strongly agree, 2—Agree, 3—Neither agree nor disagree, 4—Disagree, 5—Strongly disagree.

***Shipping-related concerns and preferences***					
Free shipping makes me more likely to buy something online.	1	2	3	4	5
I worry that my online order might not be delivered.	1	2	3	4	5
I worry that I might get the wrong product when I shop online.	1	2	3	4	5
I worry that clothes I order online might not fit.	1	2	3	4	5
I worry that products I order online might not be fresh or good quality.	1	2	3	4	5
I can choose weekend delivery when I shop online.	1	2	3	4	5
***Price sensitivity and perceived cost advantage***					
I think online shopping helps me save money.	1	2	3	4	5
I spend less money per purchase online than in stores.	1	2	3	4	5
I spend less per order when I shop online compared to in stores.	1	2	3	4	5
***Quality perception***					
I think the quality of products I buy online is similar to what I buy in stores.	1	2	3	4	5
I get similar buying conditions online as I do in physical stores.	1	2	3	4	5
The products I order online usually match what I see on the website.	1	2	3	4	5
***Security concerns***					
I feel uncomfortable entering my card details when shopping online.	1	2	3	4	5
I am concerned about losing my privacy when I shop online.	1	2	3	4	5
I worry that my identity could be stolen when I shop online.	1	2	3	4	5
***Time-related benefits***					
I save time when I shop online.	1	2	3	4	5
I like that I can shop online at any time.	1	2	3	4	5
Online shopping is a good way for me to spend my time.	1	2	3	4	5
***Availability and quality of information***					
The product information I see online is the same as what I find in stores.	1	2	3	4	5
The product information I see online is clear and accurate.	1	2	3	4	5
The product information I see online is up to date.	1	2	3	4	5
***Shopping services***					
I am happy that I can shop online.	1	2	3	4	5
I find online shopping interesting.	1	2	3	4	5
The product information I see online is up to date.	1	2	3	4	5
I would recommend online shopping to others.	1	2	3	4	5
I enjoy buying things online.	1	2	3	4	5
I think online shopping is a good way to shop.	1	2	3	4	5
